# Toxicological Assessment of Ketamine in Juvenile Zebrafish (*Danio rerio*)

**DOI:** 10.3390/toxics13020082

**Published:** 2025-01-24

**Authors:** Yin Tang, Kang Yang, Jintao Xu, Yangkai Qiu, Liang Meng, Sen Zhao

**Affiliations:** 1 Key Laboratory of State Forestry and Grassland Administration on Wildlife Evidence Tecthnology, Nanjing Police Institute, Nanjing 210023, China; tangy@nfpc.edu.cn; 2 Xi’an Forensic Science & Technology Center, Xi’an 710038, China; wozouludaifeng@sina.cn; 3 Jiashan County Public Security Bureau, Jiashan 314100, China; z10442367@gmail.com; 4 Key Laboratory of Drug Prevention and Control Technology of Zhejiang Province, Zhejiang Police College, Hangzhou 310053, China; qyk1459174135@163.com; 5 Department of Forensic Science, Fujian Police College, Fuzhou 350007, China

**Keywords:** ketamine, behavior, toxicity, zebrafish, UPLC-LTQ/Orbitrap HRMS

## Abstract

This study investigates the toxic effects of ketamine on juvenile zebrafish, driven by increasing concerns over ketamine’s prevalence and its potential neurotoxic effects that may disrupt behavior and metabolism. Employing a high-throughput behavior tracking system, the research analyzed the locomotor activity of 6-day post-fertilization (6 dpf) zebrafish exposed to various concentrations of ketamine. The integration of behavioral analysis with metabolic profiling was a notable innovation, as it establishes a comprehensive understanding of ketamine’s effects on both acute behavioral inhibition and metabolic responses. The findings reveal that ketamine exposure significantly inhibits locomotor activity in juvenile zebrafish, with these effects becoming more pronounced at higher concentrations. Additionally, the detection of normethketamine, the primary metabolite of ketamine, using UPLC-LTQ/Orbitrap HRMS, confirms the zebrafish’s ability to metabolize the drug. This underscores the utility of zebrafish as a model organism for studying the impact of ketamine on behavior and metabolism, providing valuable insights that may extend to other vertebrates.

## 1. Introduction

**Figure 1:**
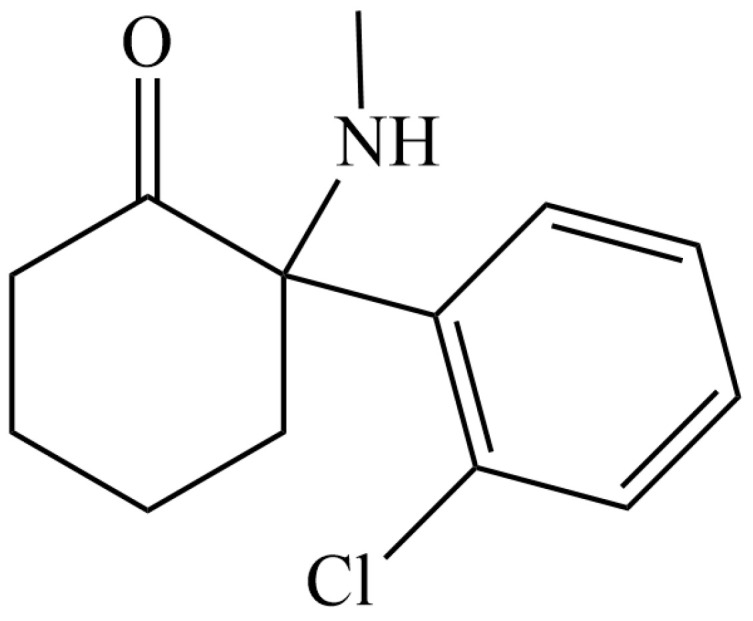
Structural formula of ketamine.

Ketamine, commonly known as “K-powder”, is chemically identified as 2-o-chlorophenyl-2-methylamino-cyclohexanone [1]. The molecular formula of ketamine is shown in Figure 1. It is a non-barbiturate intravenous anesthetic with potent analgesic properties [2]. Widely used in pediatric anesthesia, ketamine acts on synaptic sites within the central nervous system by modulating ion channel efficiency, suppressing excitatory signaling, and inducing anesthesia [3]. However, concerns about its neurotoxic effects have increasingly come to light, revealing ketamine as a substance capable of inducing strong psychological dependence, leading to its classification as a new narcotic drug [4,5]. In biological samples, ketamine’s primary metabolite, norketamine, is often co-detected. The prevalence of ketamine abuse has escalated rapidly in recent years, posing significant public health challenges. In response to this growing issue, China placed ketamine under Category I of psychotropic substances regulation as early as 2004. Thus, research on the behavioral effects of ketamine and the analysis of its metabolites is critically important to understand its broader impact on human health [6,7].

Following the consumption of drugs by individuals, the active ingredients and metabolites are excreted into urban drainage systems through urine and feces [8,9]. Additionally, these substances can enter the environment through direct disposal or discharge from illegal underground laboratories. Conventional sewage treatment processes have been found to be inadequate in fully degrading a variety of chemical contaminants, including illicit drugs, resulting in their persistence in treated wastewater and eventual discharge into surface water, drinking water, and groundwater systems [10,11]. Since 2005, there has been growing concern worldwide about the environmental risks posed by such contaminants, with numerous studies documenting the presence of substances such as ketamine, methamphetamine, and novel psychoactive substances (NPS) in wastewater [12,13]. Concentrations of these drugs have been detected at alarming levels, sometimes reaching milligrams per liter [14]. Even more concerning is the increasing evidence that these compounds have been identified in drinking water, suggesting a significant risk to both public health and the environment [15,16]. This highlights the urgent need to enhance the sensitivity and accuracy of water pollution detection methods, as well as to better understand the mechanisms through which these pollutants exert toxic effects on aquatic organisms. In this regard, zebrafish have emerged as an invaluable model organism for environmental toxicology studies. Their inherent sensitivity to environmental stressors makes them an ideal subject for assessing the harmful impacts of exogenous chemicals. Moreover, zebrafish are particularly useful for elucidating the metabolic pathways and tissue distribution of contaminants, providing critical insights into the fate and bioaccumulation of pollutants within aquatic ecosystems [17].

Zebrafish (*Danio rerio*) exhibit significant physiological similarities to humans, particularly in organ systems such as the cardiovascular, hematological, digestive, hepatic, renal, and visual systems, making them a valuable vertebrate model for biomedical research [18,19]. Due to the ease of their care, small size, rapid reproduction, and transparency, zebrafish provide a practical and efficient model for experimental observation [20]. Additionally, zebrafish offer multiple drug administration routes, including injection and immersion in pharmaceutical baths, requiring only minimal quantities of the test compound [21,22]. These factors contribute to the cost-effectiveness of zebrafish-based models, as large populations can be maintained in relatively small spaces, allowing for high-throughput studies with significant sample sizes. Consequently, zebrafish are well-suited for large-scale experimental analyses, facilitating the generation of more comprehensive and reliable data [23].

In this study, zebrafish (*Danio rerio*) were employed as a biological model to investigate the behavioral effects and metabolic processing of ketamine [24,25]. Juvenile zebrafish, serving as an analog to pediatric subjects, were exposed to ketamine to assess its impact on behavior, responsiveness to environmental changes, and adaptability. The experiment specifically aimed to analyze how ketamine influences movement patterns and external stimuli response in young zebrafish [26]. Ultra-Performance Liquid Chromatography coupled with Linear Ion-Trap/Orbitrap high-resolution mass spectrometry (UPLC-LTQ/Orbitrap HRMS) was utilized to detect and quantify ketamine and its primary metabolite, norketamine, following drug exposure via immersion [27]. The findings from this study provide valuable data for future zebrafish-based behavioral and metabolomic research, offering insights into ketamine’s potential effects on human behavior and metabolism [28,29].

## 2. Materials and Methods

### 2.1. Reagents and Consumables

A ketamine standard solution (1 mg/mL) and a norketamine standard (both obtained from Cerilliant, Merck KGaA, Darmstadt, Germany) were utilized in this study. Methanol and acetonitrile (≥99.8%), used as chromatographic-grade reagents, were sourced from Sigma-Aldrich (St. Louis, MO, USA). E3 culture medium and methylene blue solution were purchased from Beijing Chemical Reagent Company. The ketamine standard was evaporated to dryness under nitrogen gas, then diluted with E3 culture solution to the desired working concentration and stored at −20 °C for later use.

The following equipment and materials were used in this study: an ultra-high-performance liquid chromatography system coupled with a quadrupole-electrostatic field orbital trap high-resolution mass spectrometer (UPLC-Q-Orbitrap HRMS; Thermo Fisher, Waltham, MA, USA), a SORVALL ST16 high-speed centrifuge (Thermo Fisher, Waltham, MA, USA), pipettes (Thermo Fisher, Waltham, MA, USA), a C18 column (2.1 × 100 mm, 1.8 μm; Waters Corporation, Milford, WC, USA), and a zebrafish independent breeding unit (Hangzhou Newlan Technology Co., Ltd., Hangzhou, China). Additionally, a Lab Tower pure water preparation system (Thermo Fisher, Waltham, MA, USA) and a JXFSTPRP-CL sample freezing and grinding instrument (Shanghai Jingxin Company, Shanghai, China) were utilized. General laboratory consumables, including 96-well plates, plastic dropper pipettes, test tubes, and pipette tips, were sourced from Axygen (Hangzhou, China).

### 2.2. Detection Conditions of UPLC-LTQ/Orbitrap HRMS

The chromatographic separation was performed using a C18 column (2.1 × 100 mm, 1.8 μm; Waters, Milford, WC, USA). The mobile phase consisted of two components: mobile phase A, which was 1% formic acid in water, and mobile phase B, which was 1% acetonitrile in water. The column temperature was maintained at 40 °C, with a flow rate of 0.3 mL/min and an injection volume of 3 μL. Gradient elution was applied with the following parameters: 0–1 min, 99% mobile phase A and 1% mobile phase B; 7–8 min, mobile phase A decreased from 99% to 1% and mobile phase B increased from 1% to 99%; 9–10 min, mobile phase A increased from 1% to 99% and mobile phase B decreased from 99% to 1%.

The analysis was conducted using full-scan mode combined with data-dependent MS/MS (Fullscan + ddms^2^), where primary full scans automatically triggered secondary mass spectrometry scans. The scan range was set from 150 to 2000 m/z, with a resolution of 35,000 dpi. The sheath gas pressure (N_2_) was 36 arb, and the auxiliary gas pressure (N_2_) was 15 arb. The spray voltage was 3.82 kV, with a spray current of 1.20 μA. The ion transfer tube temperature was maintained at 320 °C, and the gas heater temperature was set at 289 °C.

### 2.3. Establishment of Zebrafish Toxicity Model

Wild-type (WT) AB strain zebrafish, sourced from the Wuhan National Zebrafish Resource Center, were used for the experiments. Large male and female zebrafish were selected during the months of July and August and paired in a 1:1 ratio (two males and two females). The fish were kept in a constant temperature incubator at 28.5 °C (purchased from Shanghai Longyue Instrument Equipment Co., Ltd., Shanghai, China). Fertilized eggs were collected, treated with methylene blue solution for sterilization, and labeled according to time and strain. The eggs were incubated at 28.5 °C, with screenings every 4 h to remove impurities. During the experimental procedures, all zebrafish well plates were washed with deionized water, sterilized with alcohol, and air-dried for preservation.

Based on previous domestic and international studies investigating the toxicological mechanisms of ketamine using zebrafish models [30], the maximum ketamine concentration in this experiment was set at 30 μg/mL. A stock solution of 30 μg/mL ketamine was prepared by dissolving nitrogen-dried ketamine in E3 culture medium. The tolerance of zebrafish to drugs increased with the number of survival cycles. At 6 days post-fertilization (6 dpf), juvenile zebrafish were exposed to varying concentrations of ketamine. These zebrafish were transferred to a 96-well plate for behavioral testing, where their movements were recorded in real-time using high-definition cameras positioned above the observation chamber. The EthoVision XT14 analysis system was employed to assess and analyze the behavioral data. The impact of ketamine on zebrafish behavior was evaluated by comparing the experimental group, which received the drug, with the blank control group.

Following exposure, juvenile zebrafish were collected, rinsed with deionized water, and transferred to 2 mL grinding tubes for tissue homogenization. Each tube received 1.5 mL acetonitrile and four grinding beads (3.5 mm in diameter). Samples were homogenized under low-temperature conditions for 90 s at a frequency of 60 Hz. After homogenization, the samples were centrifuged at 12,000 rpm for 10 min. The supernatant was collected and subjected to analysis using ultra-performance liquid chromatography coupled with a linear ion trap and Orbitrap high-resolution mass spectrometry (UPLC-LTQ/Orbitrap HRMS).

### 2.4. Zebrafish Sample Preparation

In this experiment, seven ketamine solutions with concentrations of 1.0 μg/mL, 2.0 μg/mL, 5.0 μg/mL, 10.0 μg/mL, 15.0 μg/mL, 20.0 μg/mL, and 30 μg/mL were prepared, along with a blank control group. A total of 120 juvenile zebrafish, aged 6 days post-fertilization (6 dpf), with healthy growth and consistent development, were selected. The fish were distributed into 96-well plates, with 12 zebrafish assigned to each concentration group. The plates were then placed into a zebrafish behavior-tracking system for observation. Each experimental cycle consisted of alternating light and dark conditions for 10 min each, designed to stimulate the zebrafish’s movement. A total of six experimental cycles were conducted.

To assess the effects of various concentrations of ketamine on the growth and development of zebrafish larvae, observations were made for signs of deformities and mortality. The ketamine solution in the original 96-well plates was replaced with an equivalent volume of E3 culture medium. The plates containing juvenile zebrafish were then placed under a fluorescence inverted microscope for detailed examination. Using CellSens (1.0) software, images of the zebrafish larvae were captured and recorded for further analysis.

### 2.5. Mass Spectrum Data Processing and Analysis

The obtained raw data files from UPLC-LTQ/Orbitrap HRMS underwent preprocessing using the Thermofisher-provided Compound Discoverer software (version CD 3.1.0.305). Differential metabolites were selected based on high-resolution identification (ppm ≤ ±5) [31] using UPLC-LTQ/Orbitrap HRMS. To ensure accuracy, Mass Frontier (3.1) software was also employed to generate potential fragments and cleavage pathways, and mass spectrometry data allowed for a comparison between actual fragment ions and those predicted by Mass Frontier. The molecular structures and ddMSMS spectra were integrated into Compound Discoverer as reference standards, while differential spectra from experimental and control groups helped identify fragment ions associated with metabolic products. This comparison enabled the identification of metabolic reaction types and sites, providing insights into the structural details and complex metabolic pathways of the metabolites. To assess the metabolites resulting from ketamine on the metabolic pathways of zebrafish, biomarkers were subjected to analysis using the MetaboAnalyst online platform.

## 3. Results

### 3.1. Effects of 6 dpf on the Mobility of Juvenile Fish

**Figure 2:**
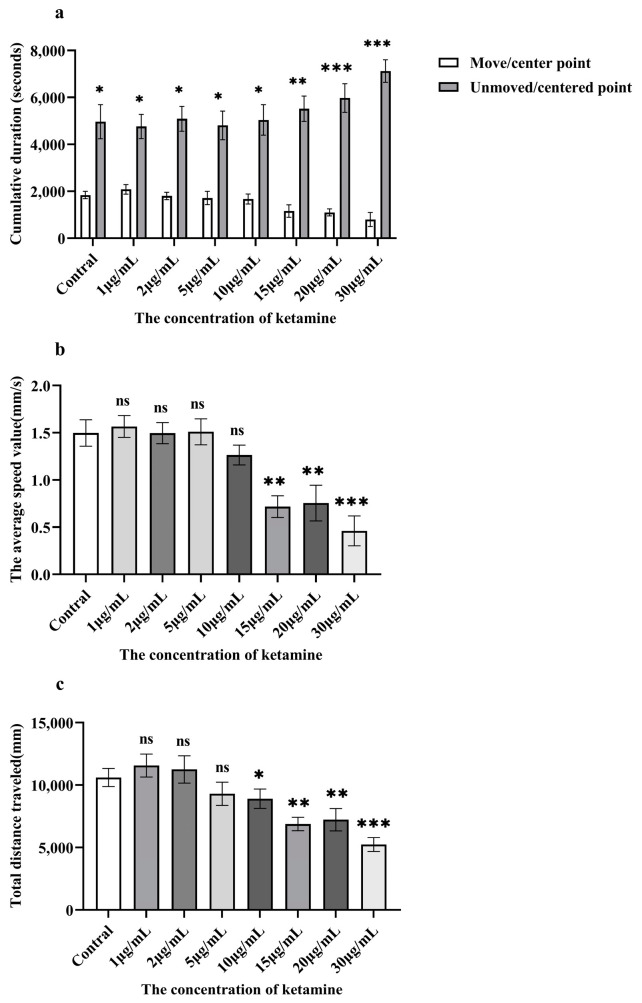
Analysis of movement parameters of 6 dpf juvenile zebrafish with different concentrations of ketamine solution. (**a**) The duration of time spent at the mobile central point. (**b**) The average swimming speed of the juvenile zebrafish. (**c**) The Total distance traveled. The results of this comparison were expressed as the mean ± standard deviation (SD) for a sample size of n = 12. Statistical significance was determined using two-tailed tests, with ns *p* > 0.05 indicating no significant difference, * *p* < 0.05 denoting a significant difference, ** *p* < 0.01 indicating a highly significant difference and *** *p* < 0.001 representing a very highly significant difference.

The data showed that with continuous exposure to ketamine, the duration of time spent at the mobile central point (Figure 2a) consistently decreased, while the time spent at the non-mobile central point increased with rising ketamine concentrations. Additionally, movement towards the central region of the environment diminished as the concentration increased, indicating a dose-dependent inhibitory effect of ketamine on the motor behavior of juvenile zebrafish.

Furthermore, the average swimming speed of the juvenile zebrafish (Figure 2b) decreased as ketamine concentration increased, with a similar trend observed for the total movement distance (Figure 2c). In the higher concentration groups (≥15.0 μg/mL), both movement speed and distance were significantly reduced, with the most pronounced inhibitory effects observed at the highest concentration (30 μg/mL).

### 3.2. Effect of 6 dpf Juvenile Fish Movement Speed Under Light and Dark Conditions

The alternating light and dark conditions effectively stimulated the motor behavior of zebrafish larvae, facilitating the observation of experimental phenomena. In this experiment, a 10-min light and dark interval was implemented, cycling through a total of six groups, with the experimental analysis chart distinguishing between the pre- and post-experimental phases at a 1-h time point. The activity of juvenile zebrafish was found to be significantly greater under dark conditions compared to light. The average movement speed of juvenile zebrafish during each 10-min light condition served as the evaluative metric for assessing their responsiveness to light and changes in behavioral adaptability.

Following the initial 10 min of exposure to bright light, the juvenile zebrafish entered the dark phase for the first time. Notably, the activity of fish in higher ketamine concentration groups (≥15.0 μg/mL) was markedly affected, exhibiting pronounced behavioral inhibition, while the other groups, including the normal control, showed no significant effects. After several cycles of alternating light and dark exposure, the movement of juvenile zebrafish in the dark was consistently lower than in the preceding cycles for those exposed to ketamine. In contrast, the control group did not exhibit a significant reduction in movement after the second light exposure, indicating that ketamine exposure diminished the zebrafish’s ability to respond to environmental changes and reduced their overall behavioral activity.

**Figure 3:**
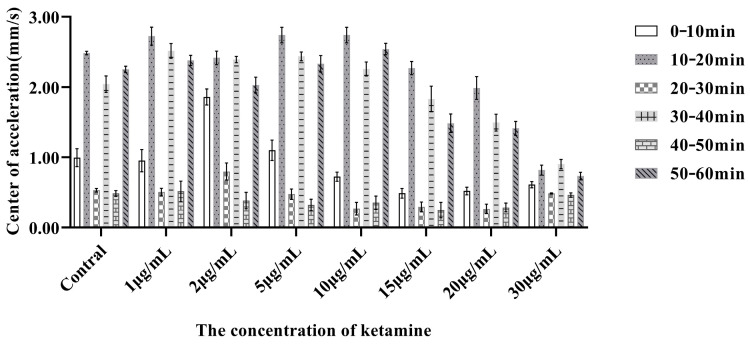
Parameter analysis of movement velocity of 6 dpf juvenile zebrafish.

Analysis of movement ability before and after the experiment at the same concentrations revealed a gradual decline in activity within the dark environment, exhibiting a stepped distribution pattern (Figure 3).

Under the first and third lighting conditions, juvenile zebrafish in the 2.0 μg/mL ketamine group exhibited significantly higher movement speeds compared to the normal control group under the first and fourth lighting conditions (Figure 3). Throughout the experiment, there was no significant decrease in movement speed for this group relative to the control under illuminated conditions, indicating an absence of inhibitory effects on locomotor ability at low concentrations. Conversely, in the 30 μg/mL group, the movement of juvenile zebrafish exhibited minimal variation under both light and dark conditions. Notably, the excitatory response of juvenile zebrafish to the dark environment following exposure to light was significantly diminished, reflecting a decreased adaptability to environmental changes and a marked reduction in their ability to navigate the dark environment.

A 10-min light and dark interval was implemented, cycling through a total of six groups, with the experimental analysis chart distinguishing between the pre- and post-experimental phases at a 1-h time point

### 3.3. Observational Tests on Juvenile Fish Samples

**Figure 4:**
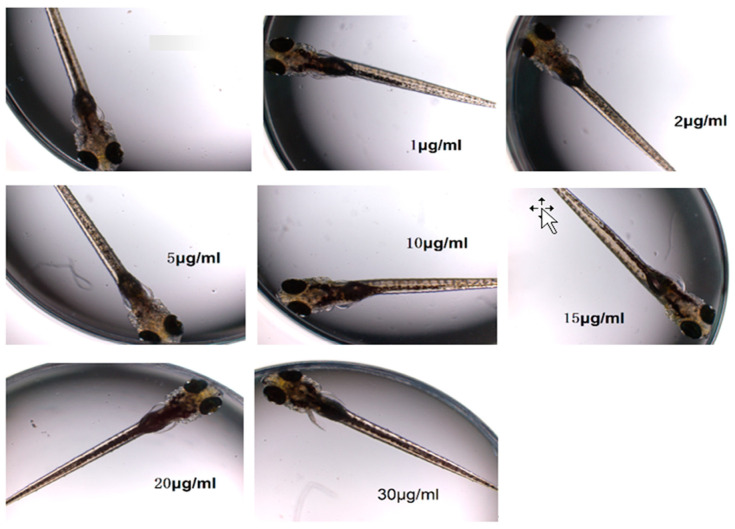
Analysis of motion velocity parameters of 6 dpf juvenile zebrafish in 1 h under different light and dark conditions (alternating every ten minutes).

Observations made using a fluorescence inverted microscope revealed that after continuous exposure of 6-day post-fertilization (6 dpf) zebrafish larvae to a low concentration of ketamine solution for two hours, there were no signs of deformities or mortality (Figure 4). The overall morphology and health of the juvenile zebrafish appeared normal. Although some differences in movement were noted, there was no direct impact on the locomotor ability of the juvenile fish.

### 3.4. Analysis of Metabolites of Ketamine

**Figure 5:**
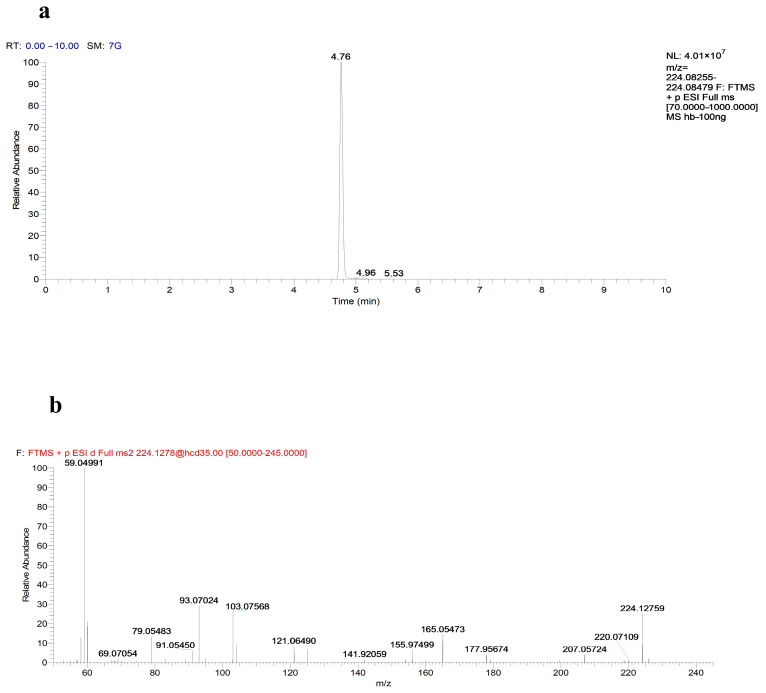
Chromatography-mass spectrum of standard norketamine samples. (**a**) The ion peaks extracted from standard norketamine samples. (**b**) The secondary mass spectrum from standard norketamine samples.

Zebrafish subjected to a medicated bath were first rinsed with deionized water, followed by grinding and extraction. The primary metabolite, norketamine, was detected using UPLC-LTQ/Orbitrap HRMS. Compound Discoverer software facilitated the analysis of metabolic pathways and the composition of metabolite spectrum fragments, with specific data presented in Table 1. Figure 5a,b illustrate the ion peaks extracted from standard norketamine samples and the corresponding secondary mass spectrum. Notably, ketamine was not detected following the grinding of zebrafish, suggesting that the experiment primarily involved a ketamine drug bath. The absorption efficiency of ketamine through the skin and gills of juvenile zebrafish appeared limited, indicating that any absorbed ketamine had been metabolized over time. However, the metabolized ketamine was sufficient to affect the zebrafish, leading to observable differences in locomotor behavior.

The primary metabolite, norketamine, was detected using UPLC-LTQ/Orbitrap HRMS, a powerful technique that provides high sensitivity and resolution for detecting low-abundance metabolites in complex biological matrices. This method, combined with the accurate mass measurement capabilities of the Orbitrap, allows for precise identification of metabolites and their structural characteristics. Compound Discoverer software was employed to facilitate the analysis of the metabolic pathways, enabling a detailed mapping of the fragment ion spectra produced during the MS/MS experiments. By analyzing the fragment ion patterns, we were able to deduce the metabolic transformations of ketamine into its primary metabolite, norketamine, and identify additional potential metabolites. The high-resolution MS data, in conjunction with Compound Discoverer’s data-mining capabilities, provided an effective means of identifying and confirming metabolic products, enhancing the understanding of ketamine’s metabolic profile and its neurotoxicological effects.

**Figure 6:**
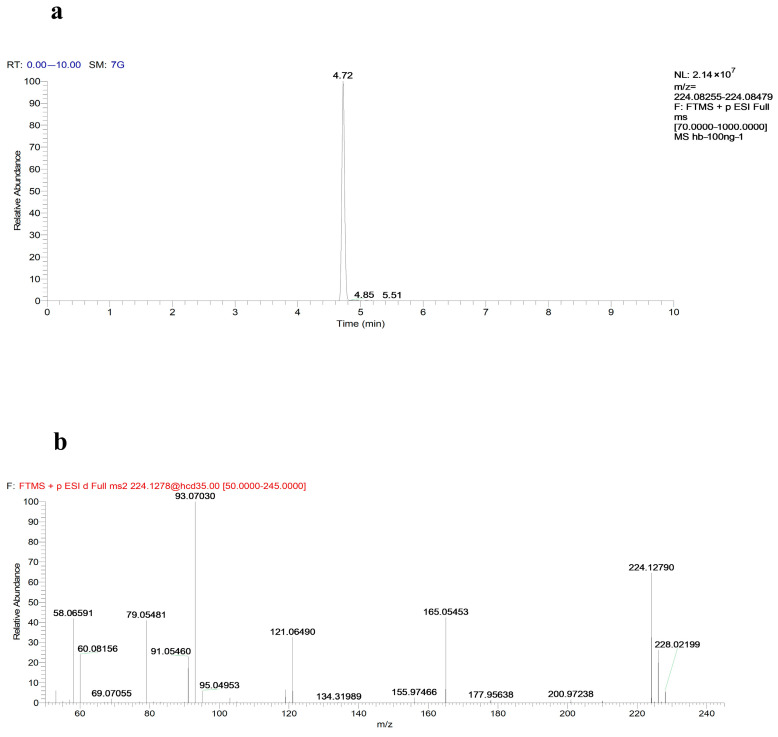
Chromatographic mass spectrometry of extracting norketamine. (**a**) The extracted ion peaks extracted from zebrafish samples. (**b**) The secondary mass spectrum data from zebrafish samples.

Following the grinding process, the extracted ion peaks for norketamine and the secondary mass spectrum data are shown in Figure 6a,b. The correlation between parent and daughter ions is detailed in Table 1, supporting the hypothesis regarding ketamine metabolism and confirming the proposed processes.

## 4. Discussion

The investigation of behavioral experimental models in neuroscience has emerged as a crucial metric for studying psychoactive substances. Zebrafish, known for their active behavior, exhibit clear responses to external stimuli, making them ideal subjects for behavioral change models [32,33]. Moreover, advancements in optical technologies and computer processing facilitate real-time observation and quantitative analysis of zebrafish behaviors following experimental manipulation, providing direct insights into behavioral alterations [34]. The experiment revealed that after exposure to ketamine, juvenile zebrafish exhibited decreased locomotor ability under alternating light conditions. Specifically, their movement capacity in dark environments significantly diminished during light–dark cycles compared to the control group. This reduced adaptability to light intensity may stem from ketamine’s inhibitory effects on the visual system, leading to diminished awareness of changes in light intensity and making it challenging for zebrafish to adjust to varying lighting conditions.

Moreover, ketamine appears to disrupt the neural circuits associated with visual processing in the zebrafish brain, further impairing zebrafish’s ability to adapt to light intensity changes. Consequently, the behavior of zebrafish exposed to ketamine under different lighting scenarios deviated markedly from that observed in normal conditions, exhibiting significant inhibition. Additionally, ketamine may alter attention and behavioral responses, resulting in anxiety-like symptoms such as confusion or restlessness in environments characterized by abrupt changes in light intensity [35]. Thus, the observed decline in zebrafish adaptability to fluctuating light intensity was likely attributable to the inhibitory effects of ketamine on their visual system and the genes involved in biorhythm perception. Further investigation into the abnormal responses elicited by drugs in relation to changes in the external environment will enhance our understanding of the toxic effects of ketamine on zebrafish.

After exposure to ketamine, zebrafish behavior demonstrated significant inhibitory effects at high concentrations (30 μg/mL) on behavioral habits, movement distance, and movement speed. Notably, these inhibitory effects became more pronounced with increasing ketamine concentration, underscoring the drug’s detrimental impact on locomotor ability. The primary consequence appeared to be a reduction in the activity level of zebrafish, leading to decreased movement distance. This inhibition may result from ketamine’s interference with energy metabolism or the neuromuscular system of zebrafish, thereby limiting both the frequency and range of their movements within the observation chamber. Such effects indicate a negative impact on zebrafish energy metabolism.

Additionally, ketamine may affect nerve conduction velocity or sensory perception related to movement, contributing to the observed decrease in swimming speed. The reduction in motor acceleration suggests that the drug disrupts neuromuscular coordination. Darland et al. utilized a T-maze to investigate the effects of cocaine on zebrafish, focusing on memory, learning, and other complex neural activities. They found that zebrafish exhibited a 60% decrease in swimming efficiency within 24 h, highlighting a continued decline in stress response [36]. Therefore, the observed reductions in behavioral indicators in this study were likely due to the direct impact of ketamine on the zebrafish nervous system. These findings provide important insights for further research into the mechanisms by which drugs influence motor behavior in zebrafish.

In this study, we employed UPLC-LTQ/Orbitrap HRMS combined with Compound Discoverer software to analyze ketamine and its primary metabolite, norketamine. While traditional techniques, like LC-MS/MS, have been widely used to detect ketamine and its metabolites, HRMS offers significantly higher resolution, accuracy, and sensitivity, making it particularly useful for detecting low-abundance metabolites in complex biological samples. HRMS allows for the precise identification and quantification of metabolites such as norketamine, and the use of Compound Discoverer software facilitated the analysis of metabolic pathways, enhancing the depth of our findings. Compared to conventional mass spectrometry, HRMS improves confidence in identifying metabolites and enables the detection of previously unreported metabolites, providing a more comprehensive understanding of ketamine’s metabolism and its impact on zebrafish.

The analysis of this experiment’s results indicates that ketamine exerts negative effects on behavior through its impact on the nervous system. These behavioral changes were attributed to ketamine and its metabolites, which alter zebrafish activity levels by influencing the concentrations of relevant neurotransmitters in brain tissue. For instance, some researchers have utilized diving tests to assess anxiety-related behaviors in zebrafish, demonstrating that exposure to tributyltin disrupts neurotransmitter pathways, thereby enhancing anxiety responses in adult male zebrafish [37]. This finding provides a valuable context for understanding the neurotoxic effects of ketamine. The observed alterations in zebrafish movement patterns were directly linked to the drug’s effects on their nervous system. The experimental results effectively illustrate how drugs can influence physiological systems at the behavioral level in zebrafish, offering important insights for future investigations into the effects of pharmacological agents on neurological function.

## 5. Conclusions

This study investigated the behavioral effects of ketamine exposure on 6-day post-fertilization (6 dpf) juvenile zebrafish. The experimental results demonstrated that exposure to ketamine resulted in stable inhibitory effects on behavioral habits, movement distance, and movement speed, with these effects becoming more pronounced at higher concentrations. Under varying light conditions, the locomotor ability of juvenile zebrafish decreased after alternating light and dark phases, with a significant reduction in movement observed in the dark environment compared to the control group. In the metabolic component of the experiment, UPLC-LTQ/Orbitrap HRMS was employed to detect the presence of normethketamine, a metabolite of ketamine, in the juvenile fish following exposure. This finding indicates that zebrafish possess the ability to metabolize ketamine, and that their behavioral responses were affected by its presence.

Although this study provides valuable insights into the effects of ketamine on zebrafish behavior, several limitations must be considered. First, the pharmacodynamic analysis of ketamine was limited to a single concentration and focused primarily on behavioral responses. A more comprehensive investigation, incorporating a range of ketamine concentrations and examining their effects across different zebrafish tissues, is necessary to better understand the drug’s systemic effects. Furthermore, the study did not explore environmental exposure scenarios, such as ketamine contamination in wastewater, which could provide critical data on the real-world risks of drug pollutants in aquatic environments. In future research, it would be beneficial to simulate such environmental exposure scenarios, measuring ketamine and its metabolites in wastewater under controlled conditions. This would enhance our understanding of the environmental fate of these substances and their potential impact on aquatic organisms. Additionally, advanced analytical techniques, including multi-omics approaches, could be employed to further elucidate the metabolic pathways and the long-term effects of ketamine on zebrafish at both the behavioral and molecular levels.

Overall, while our findings contribute to the understanding of ketamine’s effects on zebrafish behavior, there is a clear need for further studies that explore its environmental and toxicological risks more comprehensively. Future research should aim to expand the scope of pharmacological analysis, simulate realistic environmental exposure scenarios, and employ advanced technologies to assess the broader implications of ketamine and its metabolites in aquatic ecosystems.

## Figures and Tables

**Table 1:** Analysis parameters of norketamine standard and extraction.

Name	Formula	RT (min)	Mode	Theoretical Mass	Found at Mass	Mass Difference (ppm)	Characteristic Fragments
Norketamine	C_12_H_14_ClNO	4.76	ESI+	224.12767	224.12759	−0.36	93.07024, 121.06490, 165.05473
The extracted norketamine	C_12_H_14_ClNO	4.72	ESI+	224.12767	224.12790	1.02	93.07030, 121.06490, 165.05453

## Data Availability

The data that support the findings of this study are available on request from the corresponding author, Liang Meng. The data are not publicly available due to their containing information that could compromise the privacy of research participants.
